# Circulating preoperative testosterone level predicts unfavourable disease at radical prostatectomy in men with International Society of Urological Pathology Grade Group 1 prostate cancer diagnosed with systematic biopsies

**DOI:** 10.1007/s00345-020-03368-9

**Published:** 2020-07-18

**Authors:** Matteo Ferro, Giuseppe Lucarelli, Ottavio de Cobelli, Mihai Dorin Vartolomei, Rocco Damiano, Francesco Cantiello, Fabio Crocerossa, Sisto Perdonà, Paola Del Prete, Giovanni Cordima, Gennaro Musi, Francesco Del Giudice, Gian Maria Busetto, Benjamin I. Chung, Angelo Porreca, Pasquale Ditonno, Michele Battaglia, Daniela Terracciano

**Affiliations:** 1 Division of Urology, European Institute of Oncology (IEO), IRCCS, via Ripamonti 435, 20141 Milan, Italy; 2 Department of Emergency and Organ Transplantation-Urology, Andrology and Kidney Transplantation Unit, University of Bari, Piazza G. Cesare 11, 70124 Bari, Italy; 3 Department of Oncology and Hematology-Oncology, Università Degli Studi Di Milano, Milan, Italy; 4 Department of Urology, Comprehensive Cancer Center, Vienna General Hospital, Medical University of Vienna, Vienna, Austria; 5 Department of Cell and Molecular Biology, University of Medicine, Pharmacy, Sciences and Technology, Targu-Mures, Romania; 6 Department of Urology, Magna Graecia University of Catanzaro, Catanzaro, Italy; 7 Division of Urology, Istituto Nazionale Tumori di Napoli, IRCCS “G. Pascale”, Naples, Italy; 8 Scientific Directorate, Istituto Nazionale Tumori di Napoli, IRCCS “G. Pascale”, Naples, Italy; 9 Department of Urology, Sapienza Rome University, Rome, Italy; 10 Department of Urology, Stanford University Medical Center, Palo Alto, CA USA; 11 Department of Urology, Policlinico Abano Terme, Abano Terme, Italy; 12 National Cancer Institute “Giovanni Paolo II”, Bari, Italy; 13 Department of Translational Medical Sciences, University of Naples “Federico II”, 8031 Naples, Italy

**Keywords:** Prostate cancer, ISUP, Testosterone, Unfavourable disease, Upgrading, Upstaging

## Abstract

**Purpose:**

The association between circulating total testosterone (T) levels and clinically significant PCa is still a matter of debate. In this study, we evaluated whether serum testosterone levels may have a role in predicting unfavorable disease (UD) and biochemical recurrence (BCR) in patients with clinically localized (≤ cT2c) ISUP grade group 1 PCa at biopsy.

**Methods:**

408 patients with ISUP grade group 1 prostate cancer, undergone to radical prostatectomy and T measurement were included. The outcome of interest was the presence of unfavourable disease (UD) defined as ISUP grade group $\ge$ 3 and/or pT $\ge$ 3a.

**Results:**

Statistically significant differences resulted between serum testosterone values and ISUP grade groups (*P* < 0.0001). Significant correlation was found analyzing testosterone values versus age (*P* < 0.0001), and versus PSA (*P* = 0.008). BCR-free survival was significantly decreased in patients with low levels of testosterone (*P* = 0.005). These findings were confirmed also in the ISUP 1–2 subgroups (*P* = 0.01). ROC curve analysis showed that T outperformed PSA in predicting UD (AUC 0.718 vs AUC 0.525; *P* < 0.001) and was and independent risk factor for BCR.

**Conclusion:**

Our findings suggested that circulating total T was a significant predictor of UD at RP in patients with preoperative low- to intermediate-risk diseases, confirming the potential role of circulating androgens in preoperative risk assessment of PCa patients.

## Introduction

Androgens have long been recognized as “fuel” for the growth of prostate cancer (PCa) [1]. In vitro data showed that androgens caused growth of well-differentiated PCa cell lines [2], and in vivo results indicated that androgens promote prostate tumor xenograft progression [3].

Pre-operative testosterone levels association with PCa outcome is still controversial [4].

Some studies showed a significant decrease in PCa risk in men with increasing total testosterone [5]. Other authors demonstrated that high SHBG (sex-hormone binding globulin) and lower bioactive testosterone is associated with a moderate decrease in PCa risk [6].

Evidences have been reported about the association of preoperative testosterone levels and clinical outcome. In particular, circulating pretreatment testosterone levels lower than 300 ng/dL predict shorter survival and unfavourable disease [7].

At present, serum PSA, tumor grade and clinical stage are used for risk-stratification and to predict biochemical recurrence. However, there is a growing body of evidence that adding other preoperative markers may allow a more accurate prediction of disease aggressiveness, improving clinical management of PCa patient [8].

In this study, we evaluated whether serum testosterone levels may have a role in predicting unfavourable disease (UD) and biochemical recurrence (BCR) in patients with clinically localized (≤ cT2c) ISUP grade group 1 PCa at biopsy.

## Patients and methods

### Patients

This study included 544 consecutive men with localized ISUP grade 1 PCa, who underwent laparoscopic or robot-assisted radical prostatectomy (RP) within 3 months from diagnosis, between January 2009 and December 2015. Patients with known uncontrolled diabetes mellitus, endocrinopathies (i.e., thyroid disease, hyperprolactinemia), hypoalbuminemia, or liver disease were excluded (*n* = 30, 5.5%). Similarly, all patients treated with any neoadjuvant hormonal treatment throughout the previous 12 months were excluded (*n* = 34, 6.2%). A total of patients 408 (75%) were included in the final analysis.

RP specimens were processed and evaluated according to the Stanford protocol [9] by the same experienced genitourinary pathologists at each institution, blinded to the test results.

For all patients, at least 12 core biopsies were analyzed according to the 2014 International Society of Urological Pathology (ISUP) recommendations [10]. None of the study patients received neoadjuvant hormonal therapy (antiandrogens or luteinizing hormone-releasing hormone analogues or antagonists) or other hormonal preparations (i.e., 5-α reductase inhibitors) that could alter their PSA values. We also excluded patients with acute bacterial prostatitis or previous prostate surgery in the 3 months before biopsy. In addition, subjects with chronic renal disease, marked alterations in blood protein levels, hemophilia, incurable endocrine diseases or those who had previously undergone multiple transfusions, were excluded from the study because these conditions could alter the concentration of total PSA and testosterone.

Data collected included age, preoperative PSA level, PSA density, pathological stage, and preoperative serum total testosterone levels. The patients were stratified according to ISUP grade groups 1–5. Disease upstaging was regarded as pathological stage ≥ T3a after RP with clinical stage ≤ T2c. Prostate cancer upgrading was defined as ISUP grade group ≥ 3 in RP specimens.

Unfavorable disease (UD) was defined as the occurrence of pathological stage ≥ pT3 and/or ISUP grade group ≥ 3 at RP specimens pathology. Biochemical recurrence (BCR) following RP was defined according to EAU guidelines.

The threshold for hypogonadism was set at a total testosterone level of 300 ng/dL, in agreement with the American Association of Clinical Endocrinologist guidelines [11]. Accordingly, patients were further divided into two groups: (1) low total testosterone group (< 300 ng/dL) and (2) normal testosterone group (≥ 300 ng/dL).

This study received approval from the local hospital ethics committee (i.e., institutional review board approval). Written informed consent was obtained from all patients.

### Hormonal assay

All patients underwent systematic blood sampling between 7 and 10 a.m. on the day before surgery to assess serum total testosterone concentrations.

Total testosterone measurements were made at the day of sampling at different institutions using the same assay: Testosterone Elecsys II electrochemiluminescence immuno-assays (Modular Analytics E170 -Roche, Basel, Switzerland), blinded to the pathological results.

### Statistical analysis

Statistical calculations were performed with MedCalc 9.2.0.1 (MedCalc software, Mariakerke, Belgium) and PASW 18 software (PASW 18, SPSS, Chicago, Ill, USA). Comparisons of median testosterone values between different groups were evaluated by Mann–Whitney *U* test. The predictive accuracy of testosterone was evaluated using Receiver Operating Characteristic (ROC) analysis and quantified in terms of Area under the Curve (AUC) and corresponding 95% confidence interval (95% CI).

Multivariate analysis was performed using the Cox proportional hazards regression model to identify the most significant variables for predicting BCR. A backward selection procedure was performed with removal criterion *P* > 0.10 based on likelihood ratio tests. Model calibration was measured by the Hosmer–Lemeshow goodness of fit test, with *P* < 0.05 considered statistically significant.

Spearman test was applied to evaluate the correlations between testosterone levels and age, PSA, and ISUP grade groups. A *P* value of < 0.05 was considered statistically significant.

## Results

Demographic and clinical characteristics of the overall study population are summarized in Table 1.

**Table 1:** Clinical and pathological characteristics of patients

Variable	*N* = 408 Median	95% CI
Age	64	63–65
PSA	5.370	5.177–5.702
Testosterone	456	432–500
Testosterone < 300 ng/dL	151 (37%)	
PSA_density	0.120	0.120–0.130
Nr of positive cores, 2	189 (47%)	
Max % of core involved by tumor	30	20–30
Pathological stage		
pT2	363 (89%)	
pT3	45 (11%)	
ISUP grade group		
1	243 (59.5%)	
2	68 (16.7%)	
3	79 (19.4%)	
4	8 (1.9%)	
5	10 (2.5%)	

**Fig. 1:**
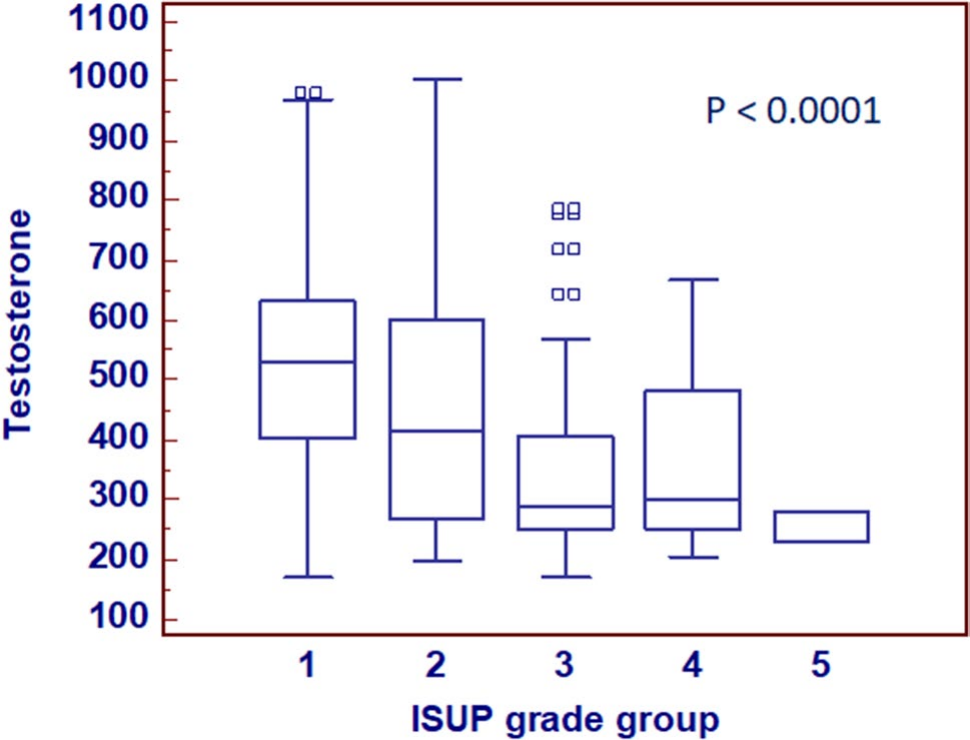
Preoperative serum testosterone levels stratified by ISUS grade groups

**Fig. 2:**
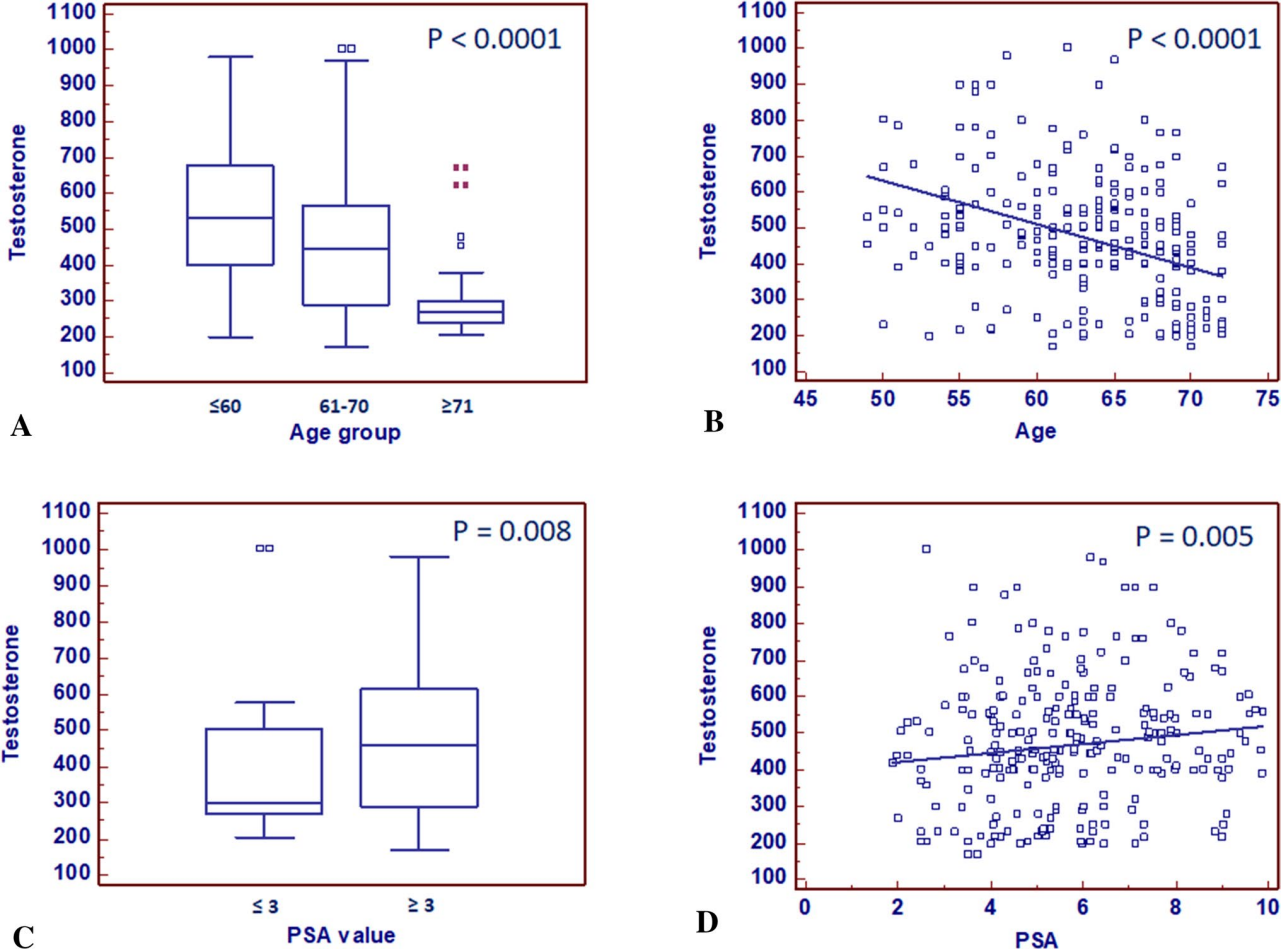
Preoperative serum testosterone levels stratified by age groups (**a**). Scatter diagram with regression line between testosterone levels and age (**b**). Preoperative serum testosterone levels stratified by total PSA groups (**c**). Scatter diagram with regression line between testosterone levels and total PSA values (**d**)

Statistically significant differences resulted between serum testosterone values and ISUP grade groups (*P* < 0.0001; Spearman correlation: rs = − 0.366, *P* < 0.0001) (Fig. 1). Significant correlation was found analyzing testosterone values versus age (*P* < 0.0001; rs = − 0.386, *P* < 0.0001), and versus PSA (*P* = 0.008; rs = 0.133, *P* = 0.005) (Fig. 2a–d).

**Fig. 3:**
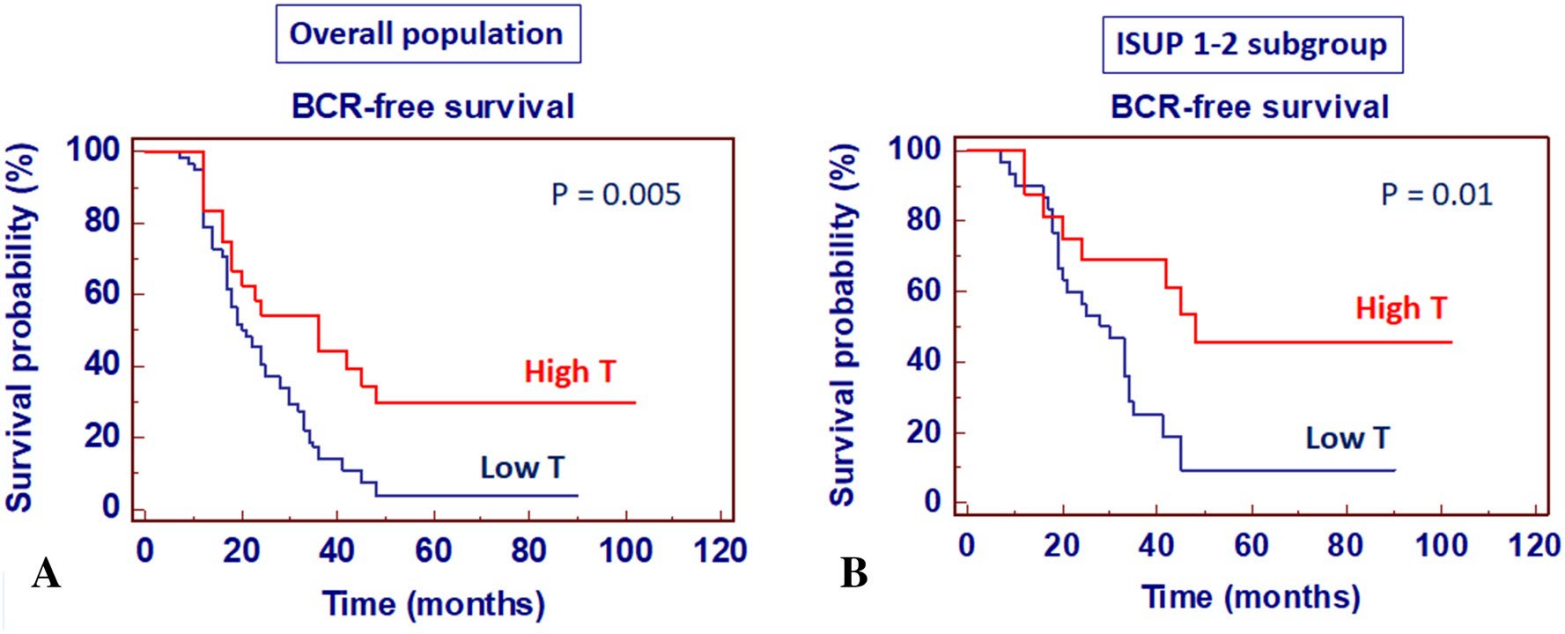
Kaplan–Meier biochemical recurrence (BCR)-free survival curves, stratified by preoperative total testosterone levels in overall population (**a**), and ISUP 1–2 subgroup (**b**)

Kaplan–Meier survival curves for BCR-free survival, stratified by serum testosterone levels for overall population, are shown in Fig. 3A. BCR-free survival was significantly decreased in patients with low levels of testosterone (*P* = 0.005). These findings were confirmed also in the ISUP grade 1–2 subgroup (*P* = 0.01) (Fig. 3b).

**Fig. 4:**
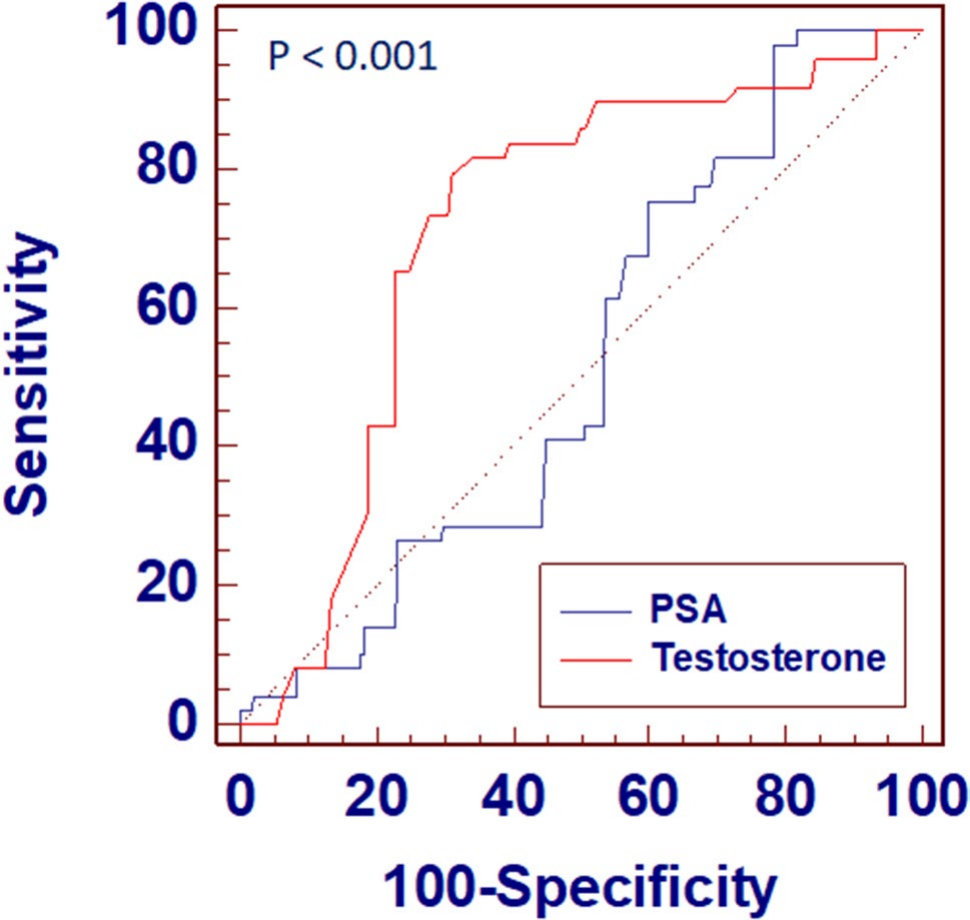
ROC curve analysis testing the accuracy of total testosterone and PSA in predicting unfavourable disease

ROC curve analysis testing the accuracy of total testosterone and PSA in predicting unfavorable disease showed that total testosterone had the best predictive values (AUC 0.718; 95% CI 0.671–0.762), outperforming PSA (AUC 0.525; 95% CI 0.475–0.575), *P* < 0.001 (Fig. 4).

Multivariate logistic regression analysis (Table 2) demonstrated that age, ISUP grade, and total testosterone were significant independent predictors of BCR, with the Hosmer–Lemeshow statistics showing adequate model calibration (*P* = 0.8).

**Table 2:** Logistic regression model results for biochemical recurrence

Variable	Odds ratio	95% CI	*P* value
Age	0.928	0.8828–0.9803	0.0058
ISUP group	2.077	1.5345–2.8071	0.0001
Testosterone (continuously coded)	1.2966	1.0949–1.9881	0.0016
PSA (continuously coded)	1.1784	0.9962–1.3939	0.0735

## Discussion

At present, clinically significant PCa is defined on the basis of preoperative PSA, clinical stage and biopsy Gleason score [12].

Prostate cancer is considered an androgen-dependent tumour and several authors showed that preoperative testosterone levels was a predictor of PCa aggressiveness [6, 7, 13, 14].

Accumulating data indicate an important association between low testosterone concentrations and worrisome aspects of PCa. Multiple studies have reported the association of lower serum testosterone values with high-grade PCa and a higher stage at presentation [15, 16]. In accordance with these results, we found a negative correlation between the preoperative testosterone levels and Gleason score (ISUP grade group).

In addition, several authors demonstrated that low pretreatment T levels are independent predictors of aggressive PCa at radical prostatectomy [13, 14]. Conversely, other studies showed that high T levels are associated with high Gleason score PCa at final pathology [17]. However, the exact relationship between total testosterone levels and clinically relevant PCa is still a matter of debate [4]. Yamamoto et al. showed that preoperative total testosterone was an independent predictor of biochemical recurrence after RP in patients with clinically localized PCa [18]. In their retrospective evaluation of 272 patients, testosterone was not associated with any perioperative clinicopathologic variables (Gleason score, pathologic stage, surgical margins). The authors indicated that the cause of these findings was unclear. More recently, other authors suggested that circulating testosterone level was a significant predictor of ISUP grade group 5 PCa at RP in patients with preoperative low- to intermediate-risk disease [19].

Since published studies present contradictory results, we evaluated the association between circulating preoperative testosterone levels and diagnosis of unfavourable disease at RP in a large cohort of patients showing ISUP 1 PCa at biopsy. Our results suggested a significant relationship between T levels and ISUP grade group  ≥ 3, extra-capsular extension, and biochemical recurrence after RP. Our analysis showed that BCR-free survival was significantly decreased in patients with low levels of testosterone and these results were observed also in the ISUP grade 1–2 subgroup. In particular, in ISUP I-II subgroup (311 patients), only 32 patients (10.2%) experienced BCR. 47% of these patients had disease upstaging and 53% had upgrading. Therefore at least half of the BCR cases observed in this subgroup, can be due to an incorrect preoperative evaluation of this population.

Our findings support the linear correlation between preoperative T and disease aggressiveness, classified by the new Epstein grading system [10]. Such a system showed a higher accuracy in the identification of poorer clinical outcome.

The biological rationale for the association of low T levels with aggressive PCa has not been well clarified yet. Some authors reported that PCa cell growth was affected by androgen only below a certain concentration [2]. Moreover, when intraprostatic androgen levels was low, the dissociation of androgen from their receptor was slower.

Furthermore, our study was focused on a cohort of low-risk patients at the time of diagnosis, so the identification of T as independent predictor of aggressive cancer may have a relevant clinical impact.

Moreover, circulating T levels decreased with age since the fourth decade of life [20–22], suggesting that T measurement may be crucial in aging.

Our study presents some limitations. Firstly, we did not use mass spectrometry for circulating T measurement, so we lack gold-standard method [23]. Secondly, as the other sex steroid hormone, T varies during lifespan of the patient [24], therefore a single T determination might not be representative of the prostate hormonal milieu. In conclusion, our results need to be validated in a larger multi-ethnic study population, allowing to better define T cut-off values to be used in clinical practice as independent prognostic indicator.

Furthermore, we did not evaluated intraprostatic concentration of androgens, which does not always mirror systemic total T levels [4]. Nevertheless, this measurement would have scarce implementation in clinical practice. Finally, our results represented a large homogeneous cohort of men, so to translate them in a real-life setting and to identify specific preoperative total T cut-off values, further studies are needed on larger study population including subjects with different ethnic origin.

## Conclusion

Our findings suggested that in patients with preoperative low- to intermediate-risk disease low circulating total T levels was a predictor of unfavourable prognosis according to the most recently proposed PCa grading system. Therefore, total T measurement may be clinically useful to identify patients with favorable preoperative disease characteristics harboring aggressive PCa, suggesting that total T may represent a tool in the treatment-decision process.
